# Effect of oncolytic ECHO-7 virus strain Rigvir on uveal melanoma cell lines

**DOI:** 10.1186/s13104-020-05068-4

**Published:** 2020-04-16

**Authors:** Andra Tilgase, Lita Grīne, Ilze Blāķe, Mārtiņs Borodušķis, Agnija Rasa, Pēteris Alberts

**Affiliations:** 1 Rigvir, Atlasa iela 7C, LV-1026 Riga, Latvia; 2 University of Latvia, Jelgavas iela 1, LV-1004 Riga, Latvia

**Keywords:** Uveal melanoma, Oncolytic virus, ECHO-7, Rigvir

## Abstract

**Objective:**

Uveal melanoma is a rare intraocular malignancy. Half of the patients diagnosed will develop metastases within 10 to 30 years, most commonly in the liver. Although there has been a significant development in the treatment of melanoma, no effective treatment to prevent or treat metastases of uveal melanoma is available. Oncolytic viruses are now being studied for various types of cancers and show promising results. Preclinical results show cytolytic activity of enteric cytopathic human orphan virus type 7 (ECHO-7) strain Rigvir in human melanoma, rhabdomyosarcoma, gastric adenocarcinoma, lung carcinoma and pancreas adenocarcinoma cell lines. The aim of this study was to test the possible cytolytic activity in human uveal melanoma cell lines.

**Results:**

The results suggest cytolytic activity of oncolytic ECHO-7 virus strain Rigvir in MP41, 92-1 and Mel-202 cell lines.

## Introduction

While uveal melanoma is a rare disease it is one of the most commonly diagnosed intraocular malignancies, which comprises about 5% of all melanoma cases reported [1]. Most of the cases, 90%, originate from the choroid, with the remaining from the ciliary body (5%) and the iris (5%) [2]. It is estimated that 50% of the patients will develop metastases, mostly in the liver [3]. Currently there is no effective way to prevent or treat metastases of uveal melanoma [4].

Cutaneous and uveal melanoma both arise from melanocytes, however, genetically they are different. In cutaneous melanoma most of the mutations concern the mitogen-activated protein kinase pathway (MAPK). This pathway is responsible for cell growth, differentiation and survival. The mutations altering this pathway most commonly are found in the serine/threonine-protein kinase B-Raf (BRAF) kinase; 40–50% of cutaneous melanoma have *BRAF* mutations [5]. In contrast, uveal melanoma most often involves the MAPK activating genes *GNAQ* and *GNA11* [6]. These genes activate the signalling between G-protein coupled receptors and upregulate MAPK pathway signalling [6]. The tumour suppressor *BAP1* gene shows inactivating mutations in 85% of aggressive tumours and is considered to be a marker of metastatic disease [7].

Due to the distinctive genetic features of uveal and cutaneous melanoma, specific model systems for each should be used in preclinical studies and active substance testing. Until recently, there was a lack of cell models for uveal melanoma research because most of the available cell lines were from cutaneous melanoma. However, recently a couple of uveal melanoma cell lines have been developed [8]. They include 7 cell lines that were established from uveal melanoma tumours or patient derived uveal melanoma tumour xenografts [8]. All of these cell lines possess the distinct genetic features of uveal melanoma.

Cytolytic effect of the present ECHO-7 virus strain has been observed in human melanoma, rhabdomyosarcoma, gastric adenocarcinoma, lung carcinoma and pancreas adenocarcinoma cell lines [9]. Currently, several oncolytic viruses are being tested for the treatment of uveal melanoma. For example, the oncolytic adenovirus ICOVIR-5 has been tested in phase I clinical trials for cutaneous and uveal melanoma. While ICOVIR-5 did reach the tumour, no effect on tumour regression was observed after a single injection, suggesting that systemic virus administration for a longer period of time should be tested [10]. Herpes simplex virus (HSV) has already been used as a treatment for cutaneous melanoma. The oncolytic potential of HSV-1 has been tested in 3D uveal melanoma cell spheroids. The results indicate that HSV-1 virus has oncolytic potential in some of the melanoma cell lines but also induce growth of other melanoma cells [11].

The oncolytic potential of Rigvir has been observed in several cancer cell lines. Therefore, the experiments were extended to examine the effect on cell viability of oncolytic ECHO-7 virus strain on uveal melanoma cell lines. The following seven cell lines were tested: Mel–202, MP41, MP38, MP65, MP46, MM28 and 92-1.

Only 3 of the seven uveal melanoma cell lines could be successfully propagated in the laboratory, Mel-202, MP41 and 92-1, and used in the analysis. The results indicate that Rigvir had cytolytic effect in Mel-202, MP41 and 92-1 uveal melanoma cell lines.

## Main text

### Methods

Cell lines MP38 (American Type Culture Collection, ATCC CRL-3296), MP65 (ATCC CRL-3299), MP41 (ATCC CRL-3297), MP46 (ATCC CRL-3298), MM28 (ATCC CRL-3295) were obtained from ATCC, and 92-1 (cat.no. 13,012,458-CDNA-20UL) and Mel-202 (cat.no. CLS3527) were obtained from Sigma-Aldrich. All cell lines are of human origin. The cell lines were cultured in Roswell Park Memorial Institute Medium (RPMI) + 20% foetal bovine serum (FBS) + 1% penicillin/streptomycin under 37 °C, 5% CO_2_ growth conditions.

ECHO-7 virus strain (Rigvir batch no. B1417R, 10^6^ TCID_50_/ml) was obtained from the manufacturer.

Inactivated ECHO-7 virus strain (Rigvir batch no. B1417R) was treated with gamma irradiation; the measured absorbed dose in the samples was 41.6 -43.3 kGy.

10,000 cells for culture 92-1 and MP41, 30,000 cells for Mel-202 were seeded with culture medium (RPMI + 20% FBS + 1% penicillin/streptomycin) per 1 cm^2^ in a 24 well plate. After 24 h cultivation (to ensure complete cell attachment to plate surface) the medium was renewed.

Two concentrations of ECHO-7 virus Rigvir strain were used: The number of viral particles added was multiplicity of infection (MOI) 7 and 70 (1% and 10% (v/v), respectively). Also, two concentrations of inactivated ECHO-7 virus strain were added: MOI of 7 and MOI of 70 as negative control. An equal volume of medium (without virus) was added to the control cells. Each test group was run in triplicate (n = 3).

Cell proliferation was monitored for 96 h with a live cell imaging system (Cell-IQ, now CellActivision, Yokogawa). Phase contrast microscopy images were taken after 0, 3, 6, 12, 24, 48, 72 and 96 h. The live cell imaging system was set to recognize differences in cell population and morphology of different cell growth phases.

The inhibitory effect of ECHO-7 virus Rigvir strain was calculated at the end of culturing time (96 h), using the formula: Inhibition (%) = 100-(100xA/B), where A stands for cell number with Rigvir at the end of culturing time, and B is control (control with phosphate-buffered saline, PBS, MOI of 7 inactivated control and MOI of 70 inactivated control, respectively) the cell number at the end of the culturing time. GraphPad Prism 8 was used for two-way ANOVA statistical analysis. The difference was taken as statistically significantly different when P < 0.05.

### Results

**Fig. 1:**
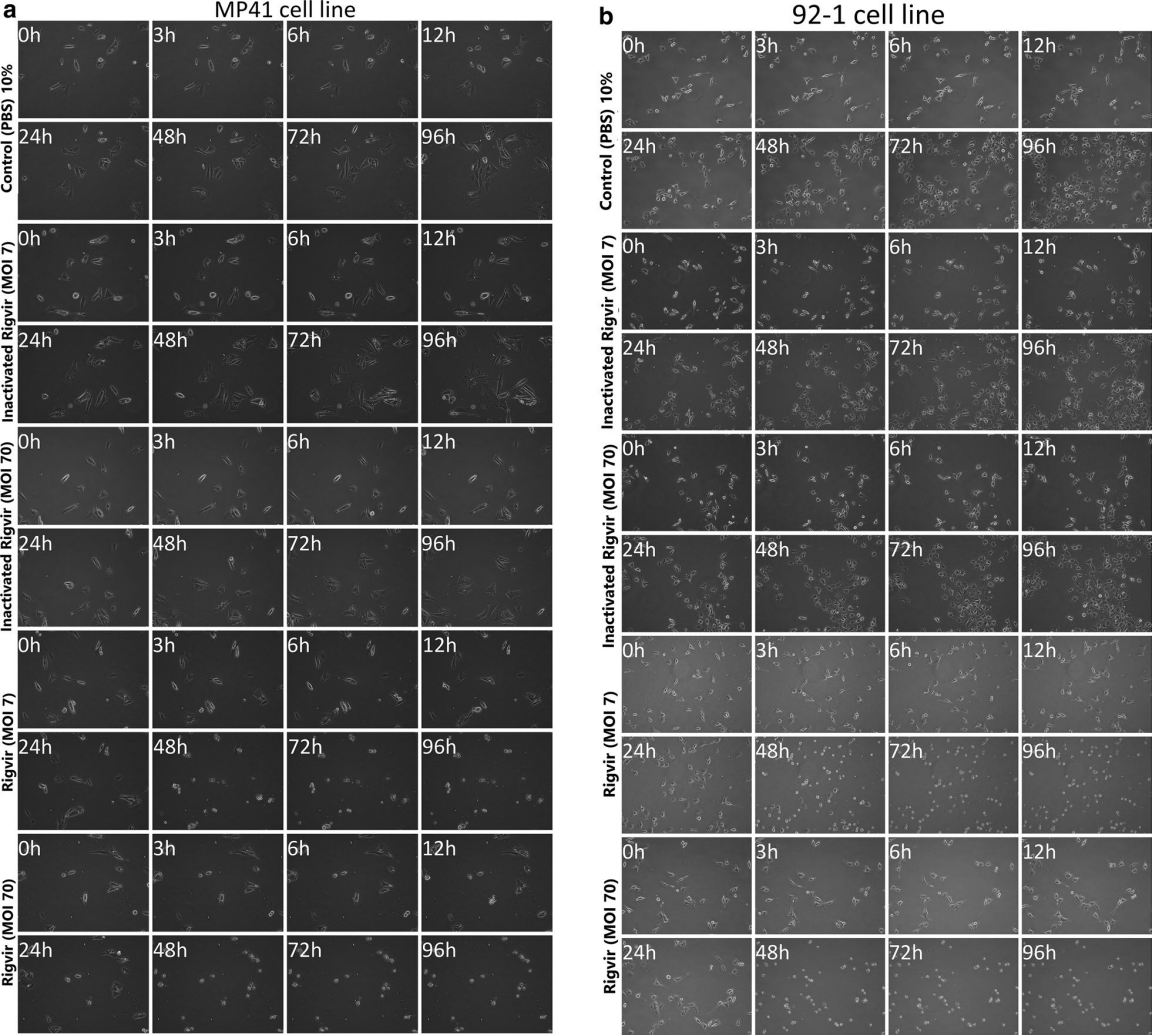

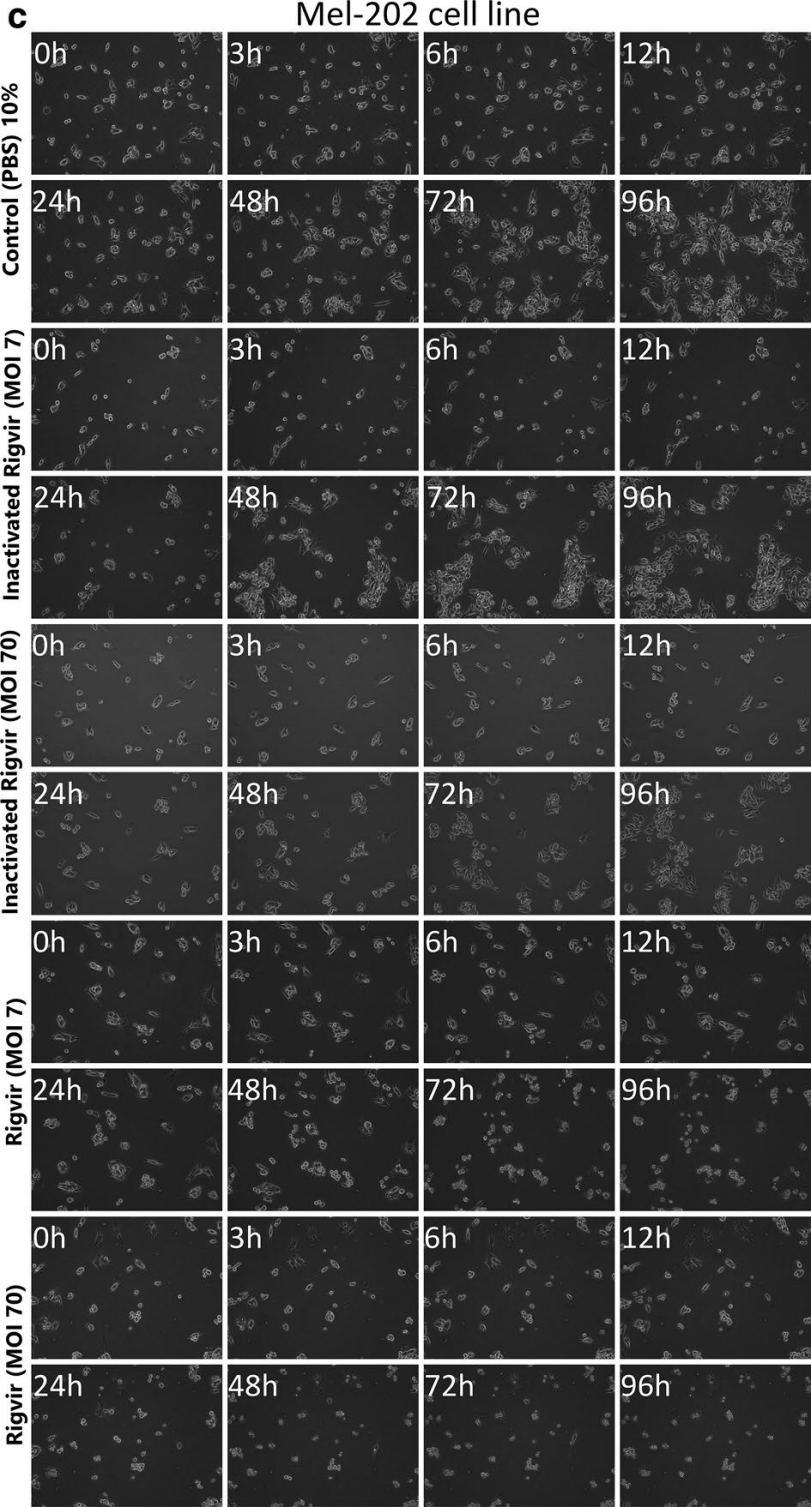
Effect of ECHO-7 virus strain Rigvir on MP41 (**a**), 92-1 (**b**) and Mel-202 (**c**) cell culture viable cell count. Phase contrast microscopy photographs taken at time points 0, 3, 6, 12, 24, 48, 72 and 96 h of incubation. From top to bottom, control (PBS), control with inactivated Rigvir (MOI 7), control with inactivated Rigvir (MOI 70), Rigvir (MOI 7) and Rigvir (MOI 70)

**Fig. 2:**
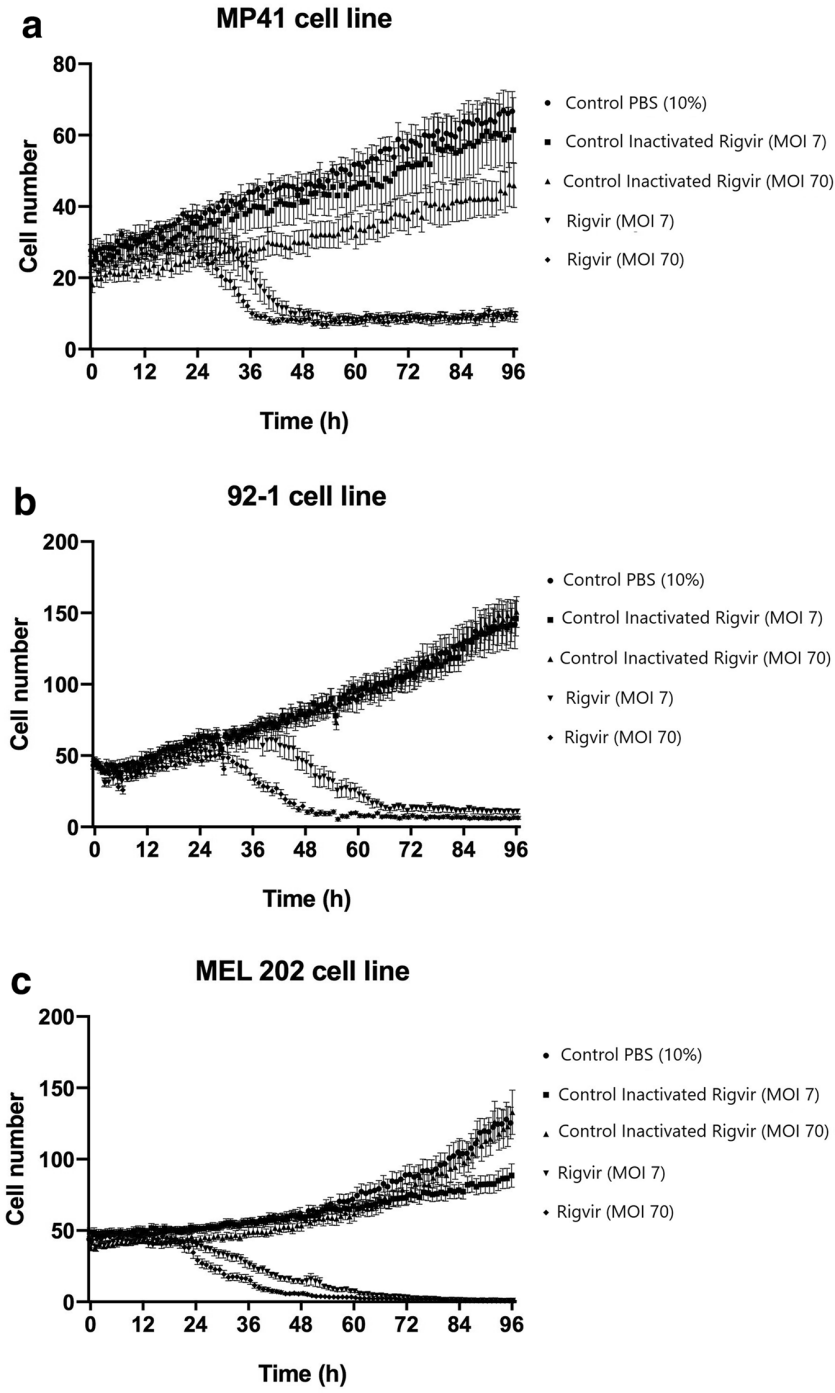
Effect of ECHO-7 virus strain Rigvir on viable cell count 0 to 96 h. Data are expressed as mean ± S.E.M. Control PBS (Black circle), control with MOI of 7 inactivated Rigvir (Black square), control with MOI of 70 inactivated Rigvir (Black up-pointing triangle), Rigvir MOI of 7 (Black down-pointing triangle) and Rigvir MOI of 70 (Black diamond). **a** MP41 cells: Statistically significant difference between Control (PBS) vs. treated with Rigvir (MOI 7) at 35 h (P = 0.0404) and Rigvir (MOI 70) at 13 h (P = 0.028). **b** 92-1 cells: Statistically significant difference between Control (PBS) vs. treated with Rigvir (MOI 7) at 49 h (P = 0.035) and Rigvir (MOI 70) at 35 h (P = 0.0013). **c** Mel-202 cells: statistically significant difference between control (PBS) vs. treated with Rigvir (MOI 7) at 24 h (P = 0.01) and Rigvir (MOI 70) at 24 h (P < 0.0001)

The results show that MP41, Mel-202 and 92-1 cell line cells detached from the cultivation surface when ECHO-7 virus Rigvir strain was added to the medium. Control samples with PBS and inactivated Rigvir continued to proliferate. This observation indicates a cytolytic/cytotoxic effect of Rigvir in these three cell lines (Fig. 1). The cell growth curves show initial viable cell count increase for all groups. The onset of the cytolytic activity of Rigvir varied between the cell lines. For MP41 cell line the cytolytic effect onset was observed after 13 h (10% Rigvir) (Fig. 2a), and for 92-1 cell line the effect was observed later, after 35 h (10% Rigvir) (Fig. 2b). In Mel-202 cell line for both MOI of 7 and MOI of 70 Rigvir sample groups the cytolytic effect and decrease in cell number was observed starting from 24 h (Fig. 2c).

MP41 cells treated with MOI of 70 Rigvir had earlier cytotoxic response than the MOI of 7 group. The maximal inhibition of cell growth by MOI of 7 Rigvir particles compared to control (PBS) was 86.4% and by MOI of 70 ECHO-7 viral particles it was 85.9% (Fig. 2a).

92-1 cells treated with MOI of 70 Rigvir particles also showed earlier cytotoxic response than for the MOI of 7 treatment group. The maximal inhibition of cell growth by MOI of 7 Rigvir compared to control (PBS) was 92.4, and by MOI of 70 it was 95.5% (Fig. 2b).

Mel-202 cell treatment with MOI of 70 Rigvir also had earlier cytotoxic response than the MOI of 7 treatment group. The maximal inhibition of cell growth by MOI of 7 Rigvir compared to control (PBS) was 99.3% and by MOI of 70 it was 99.4% (Fig. 2c).

In the present study it was not possible to propagate the MP38, MP65, MP46 and MM28 cell lines, although all the instructions provided by the manufacturer were adhered to. Although improvement in the cell lines representing features of the uveal melanoma has been made, it is still challenging to use these lines in the cell laboratory.

### Discussion

The present results suggest that of ECHO-7 virus strain Rigvir has cytolytic activity in 92-1, MP41 and Mel-202 cell lines. These cell lines originate from primary uveal melanoma tumours, possess a loss of heterozygosity of chromosome 3, and show BAP1 protein expression. The MP41 cell line has a mutation in GNA11 (c.626 A > A/T), the 92-1 cell line has mutations in GNAQ (c.626 A > T) and EIF1AX (c.17 G/A). The Mel-202 cell line has mutations in GNAQ (c. 629 G > A) and SF3B1 (c.1793 C > T) [8]. These mutations are specific to uveal melanoma as is BAP1 protein, which is considered to be a marker of metastatic disease [7]. These mutations are found later in disease progression [12]. It has also been mentioned that the combination of loss of heterozygosity of chromosome 3 and BAP1 gene mutation cause metastases [13]. The gene mutations also have a prognostic value. EIF1AX and SF3B1 are associated with good prognosis [14]. Although GNA11 and GNAQ mutations are said to occur in an equal manner in both metastatic and nonmetastatic disease and not to be of prognostic value [14, 15]. GNA11 mutations are assumed to affect melanocytes more strongly than mutations in GNAQ, despite the proteins Gα_q_ (encoded by GNAQ) and Gα_11_ (encoded by GNA11) having 90% homologous amino acid sequences [16].

The results suggest a cytolytic effect of Rigvir in the MP41, Mel-202 and 92-1 human uveal melanoma cell lines. Currently there is no treatment to prevent or treat metastasis in the uveal melanoma patients and it is crucial that new treatment for this type of cancer are being sought.

The results show that Rigvir decreases uveal melanoma cell proliferation under in vitro conditions; further experiments are necessary to elucidate if Rigvir oncolytic virus would also have clinical effect in uveal melanoma patients.

## Limitations

This study is limited by the three uveal cell lines that were propagated in the laboratory. In order to use a live cell imaging system and capture the cytolytic effect, a certain cell layer density should be reached. Four of the cell lines have long cell doubling times (up to 96 h) that limited their use in the real time imaging system. Further studies should involve 3D cell models; however, a majority of the commercially available cell lines unfortunately do not form even cell monolayers.

## Data Availability

The data associated with this study are available from the corresponding author on a reasonable request.

## Abbreviations

ECHO-7: Enteric cytopathic human orphan virus type 7
MAPK: Mitogen-activated protein kinase
BRAF: Serine/threonine-protein kinase B-Raf
HSV: Herpes simplex virus
ATCC: American type culture collection
MOI: Multiplicity of infection
PBS: Phosphate-buffered saline
RPMI: Roswell park memorial institute medium
FBS: Foetal bovine serum
